# Genomic Analysis of a Pathogenic Bacterium, *Paeniclostridium sordellii* CBA7122 Containing the Highest Number of rRNA Operons, Isolated from a Human Stool Sample

**DOI:** 10.3389/fphar.2017.00840

**Published:** 2017-11-15

**Authors:** Joon Yong Kim, Yeon Bee Kim, Hye Seon Song, Won-Hyong Chung, Changsu Lee, Seung Woo Ahn, Se Hee Lee, Min Young Jung, Tae-Woon Kim, Young-Do Nam, Seong Woon Roh

**Affiliations:** ^1^Microbiology and Functionality Research Group, World Institute of Kimchi, Gwangju, South Korea; ^2^Research Group of Gut Microbiome, Korea Food Research Institute, Sungnam, South Korea; ^3^Department of Food Biotechnology, University of Science and Technology, Daejeon, South Korea

**Keywords:** *Paeniclostridium sordellii*, pathogen, genome sequence, rRNA operon, virulence factor

## Introduction

*Paeniclostridium sordellii* was first isolated by Alfredo Sordelli in 1922 under the proposed name *Bacillus oedematis*, and was then renamed *Bacillus sordellii* in 1927 (Hall and Scott, 1927). Two years later, it was classified as *Clostridium sordellii* (Hall et al., 1929). Recently, this bacterium was reclassified as a species of the genus *Paeniclostridium*, named *P. sordellii* comb. nov. (Sasi Jyothsna et al., 2016). *P. sordellii* is an anaerobic, Gram-stain-positive, spore-forming rod bacterium with flagella. Most strains are non-pathogenic, but some strains have been associated with severe infections of humans and animals. In humans, *P. sordellii* is mainly associated with trauma, toxic shock, soft tissue skin infections, and gynecologic infections. Despite the serious consequences of infection with *P. sordellii*, treatment is difficult because of the rapid progression from recognition of the first symptoms to death (Aldape et al., 2006).

In this study, we performed whole-genome sequencing and genomic analysis of strain CBA7122 belonging to *P. sordellii*, which was isolated from the stool sample of an 85-year-old healthy female residing in the Republic of Korea. This genomic information of *P. sordellii* CBA7122 should motivate further research on related strains, which may provide new insight into the pathogenesis of *P. sordellii* toward development of new strategies for the control, prevention, and treatment of life-threatening infections.

## Materials and methods

### Strain isolation, culture, and DNA extraction

Strain CBA7122 was isolated from the stool sample of an 85-year-old healthy female by the standard dilution plating technique on modified Eggerth-Gagnon agar medium (containing per liter of distilled water: 10 g peptone, 4 g Na_2_HPO_4_, 2 g porcine gastric mucin, 50 ml sheep blood, 15 g agar) at 37°C for 24 h in an anaerobic chamber (Coy Laboratory Products) with an atmosphere of N_2_/CO_2_/H_2_ (90:5:5, by volume). Routine cultivation of strain CBA7122 was performed under the same conditions. Genomic DNA of strain CBA7122 was extracted using the QIAamp DNA extraction kit (Qiagen, USA) and QuickGene DNA tissue kit S (Kurabo, Japan), and quantified using the Quant-iT PicoGreen dsDNA Assay kit (Invitrogen, USA). The condition of extracted DNA was assessed by agarose gel electrophoresis on a 1% agarose gel.

### Genome sequencing, assembly, and annotation

The genome sequencing of strain CBA7122 was performed using a PacBio RS II sequencing platform as described previously (Kim et al., 2016). The library based on the genomic DNA of strain CBA7122 was constructed according to the manufacturer's instructions, and sequenced using a Pacific Biosciences RS II instrument. The 150,292 generated reads were filtered and assembled using the HGAP 2 protocol with default parameters in SMRT Analysis version 2.3. Gene prediction and the basic annotation for the assembled genome of strain CBA7122 were performed using the NCBI Prokaryotic Genome Annotation Pipeline (PGAP) (Tatusova et al., 2016). In brief, 16S rRNAs and 23S rRNAs were predicted using BLASTn, and 5S rRNAs and small ncRNAs were predicted using cmsearch 1.1.1. tRNAscan-SE was used to predict tRNA gene sequences. Coding sequences (CDSs) were detected using GenMarkS+. Functional gene annotations of the genome of strain CBA7122 were performed against various databases, including the catalytic families (CatFam) (Yu et al., 2009), Clusters of Orthologous Groups (COG) (Tatusov et al., 2000), NCBI reference sequence (RefSeq) (O'Leary et al., 2016), and SEED (Overbeek et al., 2014) databases. CRISPRs were confirmed using CRISPRFinder (Grissa et al., 2007). Prophage analysis was performed by PHAST (Arndt et al., 2016).

### Comparative genomic analysis

To find unique features of the genome of strain CBA7122, the genomes of the following *P. sordellii* and *Paraclostridium bifermentans* strains were selected to perform comparative genomic analysis using the NCBI genome database (http://www.ncbi.nlm.nih.gov/genome/): *P. sordellii* strains ATCC 9714^T^ (GCA_000444075.1), VPI 9048 (GCA_000444095.1), and JGS6382 (GCA_000953555.1), and *P. bifermentans* ATCC 638^T^ (GCA_000452245.1). Genome similarities between strain CBA7122 and the reference strains were determined using Orthologous Average Nucleotide Identity (OrthoANI) values, and used to reconstruct the phylogenetic tree using the Orthologous Average Nucleotide Identity Tool (Lee et al., 2016). For comparisons at the whole-genome level, the genomes of strain CBA7122 and related strains were aligned using the progressive MAUVE algorithm in the MAUVE multiple genome alignment software 2.4.0 (Darling et al., 2004). Pan-genome analysis was performed by BIOiPLUG (Chunlab, Korea).

### Virulence factor identification

To determine the virulence factors of strain CBA7122, Basic Local Alignment Search Tool (BLAST) was used with the core dataset containing information on genes associated with experimentally verified virulence factors in the virulence factor database (VFDB) (Chen et al., 2016), with the expected e-value 0.0001.

### Ethics statement

The study protocol was approved by the institutional review board of the Theragen ETEX Bio Institute (700062-20160804-JR-005-02). Before the current study, the purpose, experimental procedure, and benefits were fully explained to the participants. Oral consents were obtained from each volunteer and consent procedure was witnessed and documented on the research record.

## Results

### General genomic features of *P. sordellii* CBA7122

**Figure 1:**
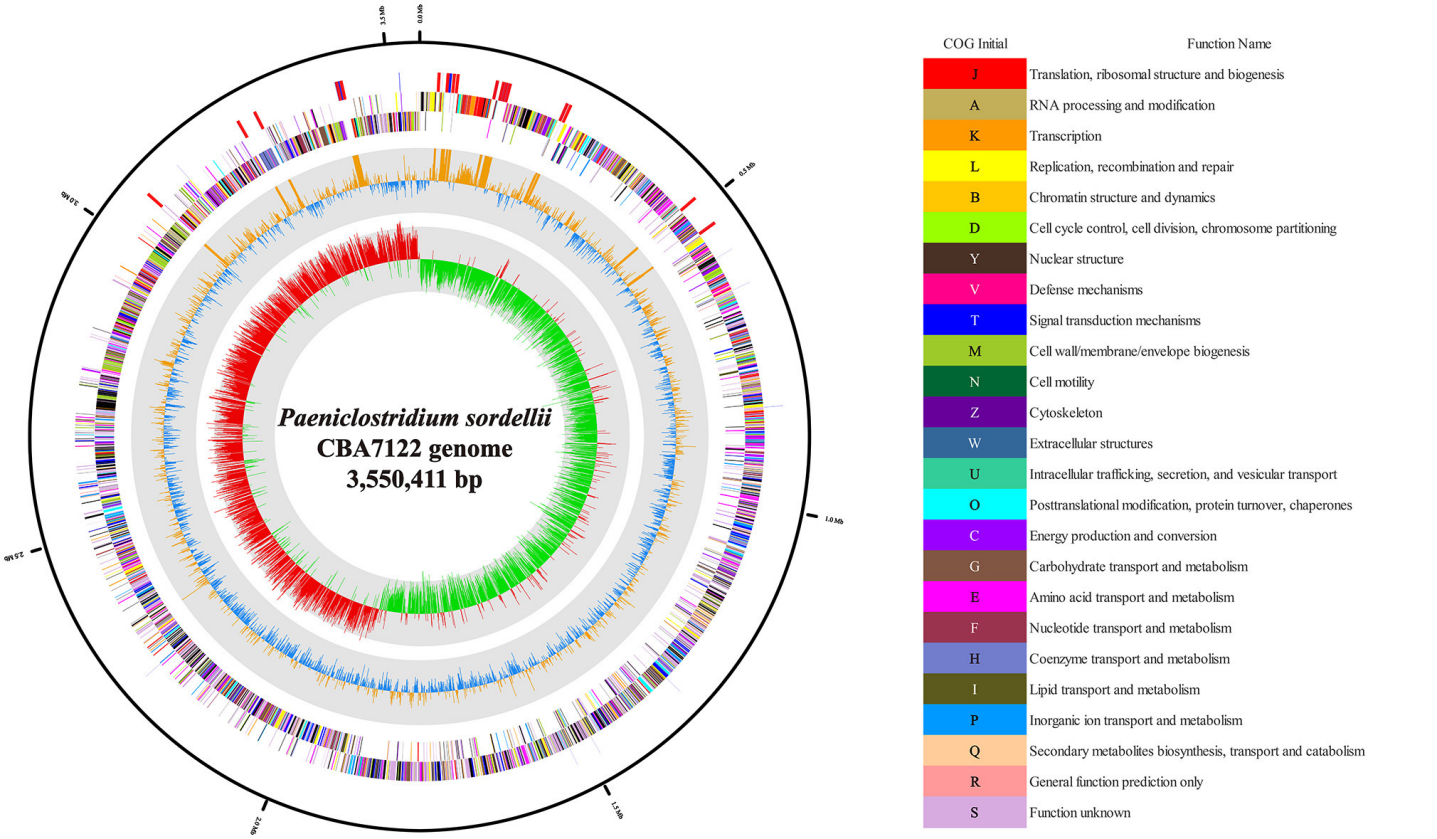
Circular map of the *Paeniclostridium sordellii* CBA7122 genome. RNA genes (red, rRNA; blue, tRNA), forward and reverse strands (colored according to COG categories) are indicated from the outer fringe to the center. Inner circles show the GC content in yellow and blue and the GC skew is shown with red and green indicating positive and negative values, respectively. This genome map was generated by CLgenomics 1.52 (Chun Lab Inc.).

The genome of *P. sordellii* strain CBA7122 comprised 3 contigs and was 3,550,411 bp long. Based on NCBI PGAP, a total of 3,459 genes were predicted, including 17 16S-23S-5S rRNA operons (51 rRNAs), 105 tRNAs, and 4 ncRNAs. The circular map of the genome is displayed in Figure 1, and detailed genome features of strain CBA7122 are listed in Table 1. Among the COG categories, 2,919 genes were categorized to transcription (230 genes); amino acid transport and metabolism (223); energy production and conversion (174); translation, ribosomal structure, and biogenesis (164); signal transduction mechanisms (156); cell wall/membrane/envelope biogenesis (155); carbohydrate transport and metabolism (153); inorganic ion transport and metabolism (143); replication, recombination, and repair (133); coenzyme transport and metabolism (93); nucleotide transport and metabolism (86); posttranslational modification, protein turnover, and chaperones (82); lipid transport and metabolism (45); cell motility (43); cell cycle control, cell division, and chromosome partitioning (33); secondary metabolites biosynthesis, transport, and catabolism (22); and function unknown (984). In the SEED database, a total of 1,470 genes were matched to the subsystem as follows: protein metabolism (264 genes); amino acids and derivatives (228); cofactors, vitamins, prosthetic groups, and pigments (206); carbohydrates (189); cell wall and capsule (169); DNA metabolism (110); nucleosides and nucleotides (105); RNA metabolism (104); virulence, disease, and defense (86); membrane transport (80); fatty acids, lipids, and isoprenoids (80); stress response (80); dormancy and sporulation (69); motility and chemotaxis (65); phosphorus metabolism (42); respiration (39); cell division and cell cycle (38); phages, prophages, transposable elements, and plasmids (28); regulation and cell signaling (25); sulfur metabolism (18); iron acquisition and metabolism (17); miscellaneous (14); potassium metabolism (11); nitrogen metabolism (9); and metabolism of aromatic compounds (1). According to CRISPR analysis, strain CBA7122 did not have known CRISPRs. Strain CBA7122 had two intact phage genomes (30,978 bp long and 41,432 bp long), located in contig 2. Furthermore, 12,687 of the 16,833-bp-long contig 1 matched to incomplete phage genes.

**Table 1:** General genome features of *Paeniclostridium sordellii* CBA7122.

**Item**	**Values**
Finishing quality	Draft
Sequencing platform	PacBio RS II
Assembler	PacBio SMRT Analysis 2.3.0
Methods reads	82,499
Genome coverage	298.06 X
Assembly size (bp)	3,550,411
N50	3,527,419
DNA G + C content (%)	27.8
Total contigs	3
Total genes	3,459
Total CDSs	3,299
RNA genes	160
rRNAs	17 rRNA operons (16S-23S-5S)
tRNAs	105
ncRNAs	4

### The number of rRNA gene copies of *P. sordellii* CBA7122

Interestingly, the genome of strain CBA7122 had 17 rRNA operons, which represented the highest number of rRNA operons known so far in the domain Bacteria. Of all *P. sordellii* genomes listed in the NCBI genome database, strain CBA7122 had the highest 16S and 23S rRNA genes copies, whereas other genomes had lower 16S (3.7 in average) and 23S (5.5 in average) rRNA genes copies (Supplementary Figure S1). In the case of the number of 5S rRNA genes, all genomes had an average of 13.7 gene copies, and strain CBA7122 had the second most number of gene copies. According to Roller et al. (2016), the number of rRNA operons is known to be associated with these two factors of reproduction, growth rate, and growth efficiency.

### Comparative genomic analysis

Based on the OrthoANI values, strain CBA7122 had greatest sequence similarity with *P. sordellii* strain JGS6382 (98.74%), followed by *P. sordellii* strains VPI 9048 (98.69%) and ATCC 9714^T^ (98.31%), and was most dissimilar to *P. bifermentans* ATCC 638^T^ (81.14%). The phylogenetic tree based on orthoANI values supported that strain CBA7122 is closely related to *P. sordellii* (Supplementary Figure S2). Whole-genome comparison of strain CBA7122 with *P. sordellii* JGS6382, VPI 9048, and ATCC 9714^T^, and *P. bifermentans* ATCC 638^T^ revealed that most of locally collinear blocks (LCBs) are closely homologous within the species rather than genus (Supplementary Figure S3). In the pan-genome analysis, a total of 4,856 pan-genome orthologous groups (POGs) were obtained from the 17,352 CDSs of the 5 genomes. As shown in Supplementary Figure S4, the core genome comprises 2,481 POGs and the genome of strain CBA7122 contains only 145 POGs as singletons. Of the 48 genes in these 145 singleton POGs, 36 were annotated to the SEED subsystem, including cell wall and capsule (8 genes); amino acids and derivatives (5); DNA metabolism (5); motility and chemotaxis (4); phages, prophages, transposable elements, and plasmids (2); carbohydrates (1); miscellaneous (1); nucleosides and nucleotides (1); phosphorus metabolism (1); RNA metabolism (1); and virulence, disease, and defense subsystem (1). The others were annotated as hypothetical or uncharacterized proteins (Supplementary Table S1).

### Virulence factors

We identified several known virulence factors in the genome of strain CBA7122, including perfringolysin O (*pfoA*), sialidase (*nanH*), thiol-activated cytolysin (*ALO*), polysialic acid capsule biosynthesis protein SiaC (*siaC*), Hsp60, 60K heat shock protein HtpB (*htpB*), capsular polysaccharide synthesis enzyme Cap8D (*cap8D*), UDP-galactopyranose mutase (*cpsI*), UDP-glucose 6-dehydrogenase (*hasB*), ATPase EscN (*escN*), glycosyl transferase CpsE (*cpsE*), *Listeria* adhesion protein Lap (*lap*), ATP-dependent protease (*clpE*), sigma 54-dependent response regulator (*fleR/flrC*), collagenase (*colA*), and UDP-galactopyranose mutase (*glf*). However, there were no large clostridial cytotoxin (LCC) genes identified in the genome of strain CBA7122, whereas these genes were identified in strains ATCC 9714^T^ and VPI 9048, which are known as the key factors of human infection leading to death.

Our data based on genomic analyses provides basic information of *P. sordellii*, which should serve as a useful reference for detailed studies focused on gaining a better understanding of the virulence factors in the genomes of these strains and their effects on human health. In addition, since strain CBA7122 contains a prophage genome, it is necessary to check whether the human infection status will be changed through phage therapy in further studies.

## Data access

The genome sequence of *Paeniclostridium sordellii* CBA7122 were deposited in the DDBJ/ENA/GenBank under accession numbers BDJI01000001–BDJI01000003.

## Author contributions

SWR and Y-DN designed and coordinated all the experiments. HSS performed cultivation, DNA extraction, and purification. JYK, YBK, SHL, and MYJ performed the sequence assembly, gene prediction, gene annotation, comparative genomic analysis, and wrote manuscript. W-HJ, CL, SWA, and T-WK checked and edited the manuscript. All authors have read and approved the manuscript.

### Conflict of interest statement

The authors declare that the research was conducted in the absence of any commercial or financial relationships that could be construed as a potential conflict of interest.
