# Supplementary material for: Genomic Analysis of a Pathogenic Bacterium, Paeniclostridium sordellii CBA7122 Containing the Highest Number of rRNA Operons, Isolated from a Human Stool Sample
Source: Front Pharmacol. 2017 Nov 15;8:840. doi: 10.3389/fphar.2017.00840 (PMC5695201; doi:10.3389/fphar.2017.00840)
Supplement: Supplementary file 1 [file DataSheet1.DOCX]

Supplementary Material

**Genomic Analysis of a Pathogenic Bacterium, *Paeniclostridium sordellii* CBA7122 Containing the Highest Number of rRNA Operons, Isolated from a Human Stool Sample**

Joon Yong Kim^1^, Yeon Bee Kim^1^, Hye Seon Song^1^, Changsu Lee^1^, Seung Woo Ahn^1^, Se Hee Lee^1^, Min Young Jung^1^, Tae-Woon Kim^1^, Young-Do Nam^2,3^* and Seong Woon Roh^1^*

^1^Microbiology and Functionality Research Group, World Institute of Kimchi, Gwangju 61755, Republic of Korea

^2^Research Group of Gut Microbiome, Korea Food Research Institute, Sungnam 13539, Republic of Korea

^3^University of Science and Technology, Daejeon 34113, Republic of Korea

***Correspondence:**

Seong Woon Roh
swroh@wikim.re.kr
Young-Do Nam
youngdo98@kfri.re.kr

# Supplementary Figures and Tables

## Supplementary Figures

**Supplementary Figure S1.** The number of rRNA operons of *Paeniclostridium sordellii* genomes. All rRNA data were obtained from the NCBI genome database (http://www.ncbi.nlm.nih.gov/genome/). Red color indicate the genes of strain CBA7122.


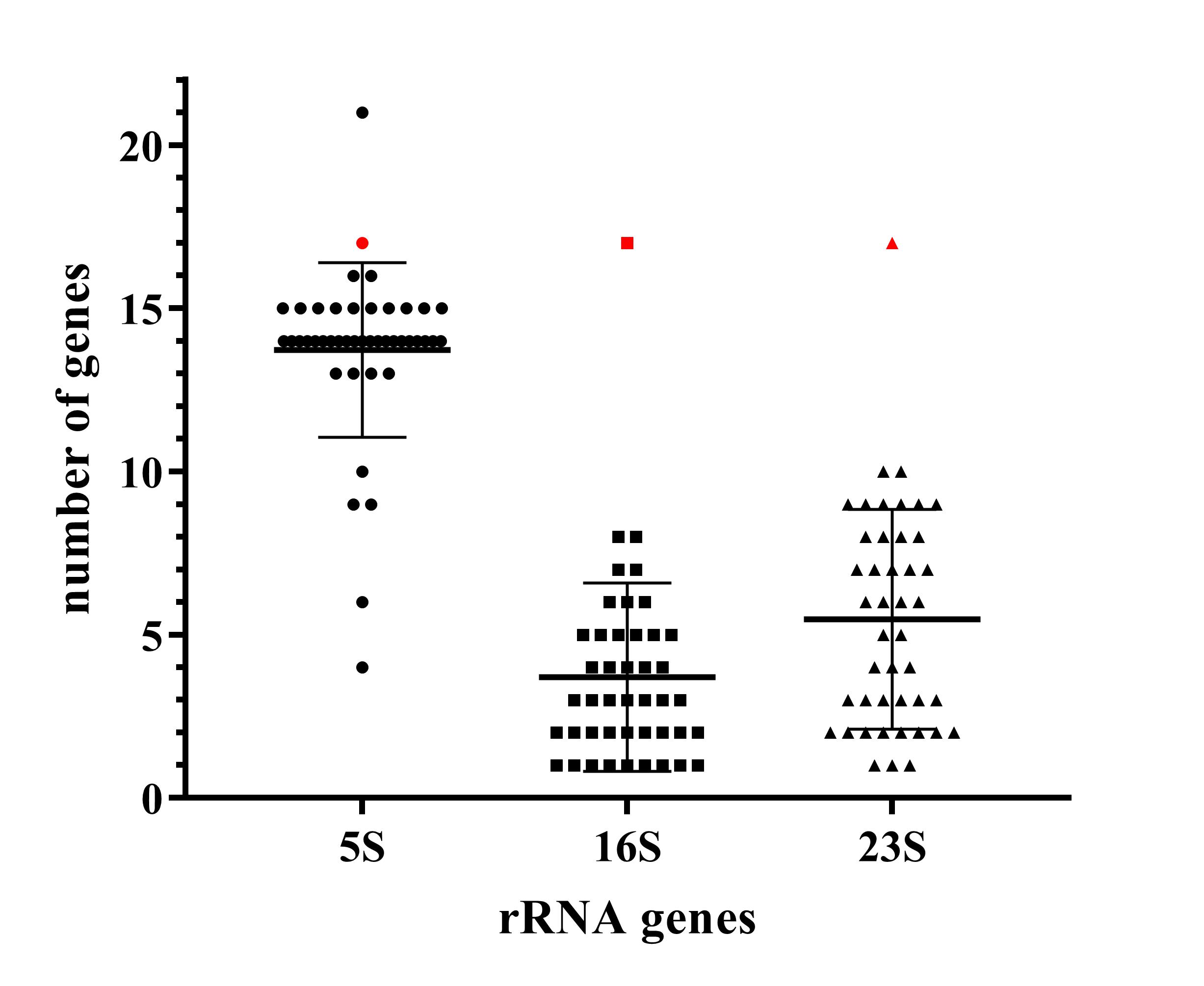


**Supplementary Figure S2.** Phylogenetic consensus trees based on the orthoANI values. The phylogenetic tree was constructed based on ANI values using BIOiPLUG.


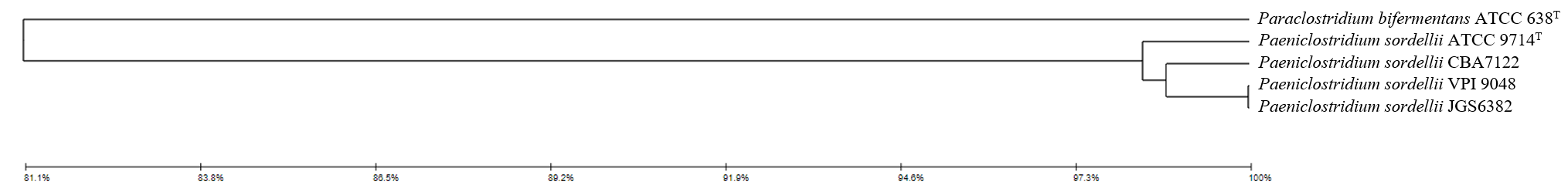


**Supplementary Figure S3.** Mauve alignment of the genome sequences of *Paeniclostridium sordellii* CBA7122 and related strains. The locally collinear blocks (LCBs) indicate highly homologous regions and are shown with identical colors. LCBs below the horizontal black line indicate reverse complements of the LCBs of the reference genomes. The genomes were drawn to scale based on the genome of strain CBA7122.


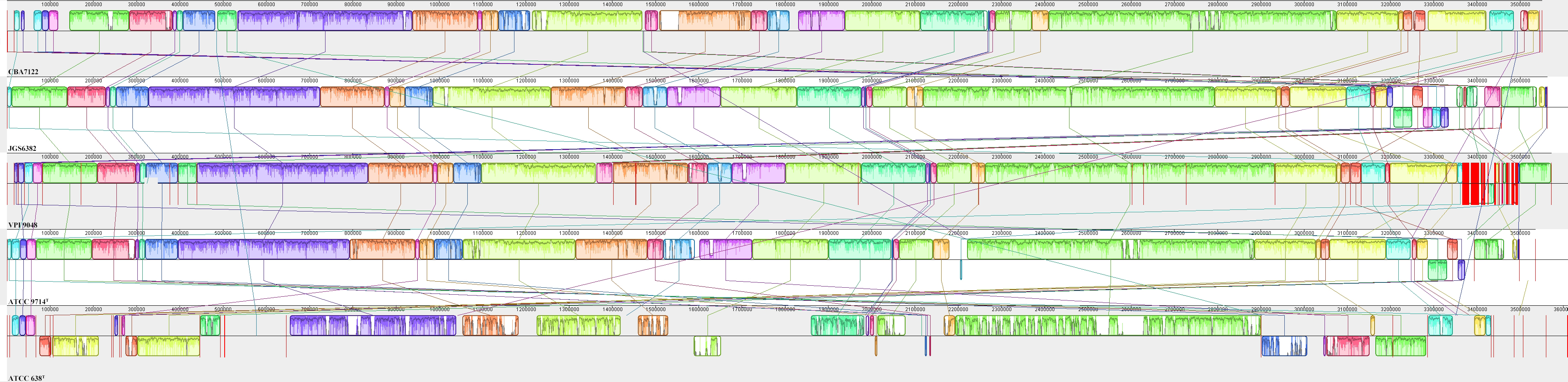


**Supplementary Figure S4**. Venn diagram representing the pan-genomic landscape of *Paeniclostridium sordellii* CBA7122 and related genomes. The numbers in the Venn diagram indicate the number of POGs found to be shared among the indicated genomes.

**
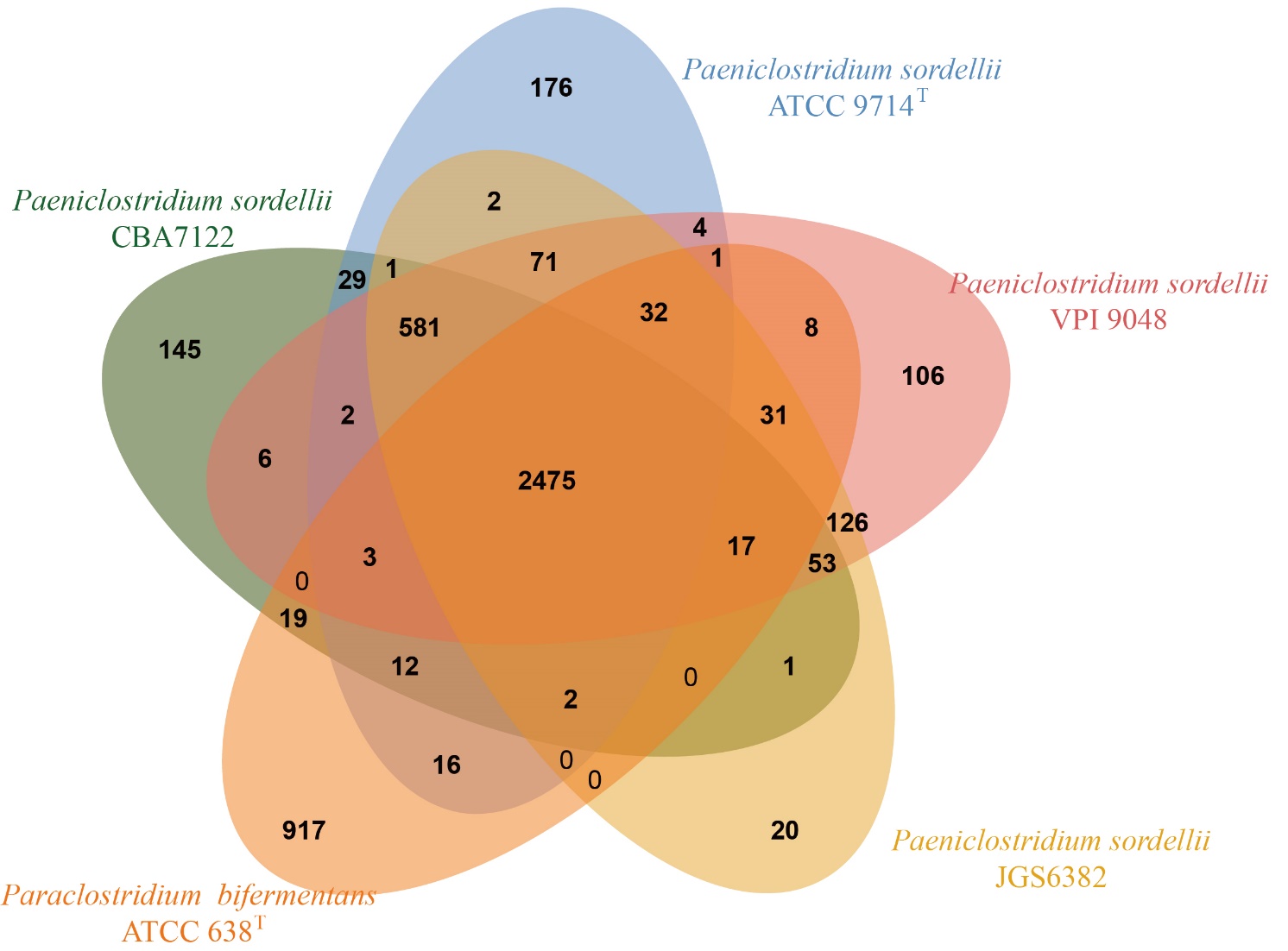
**

## Supplementary Tables

**Supplementary Table S1**. Summary of singletons of *Paeniclostridium sordellii* CBA7122.

| Subsystem | Products | Contig | location |
| --- | --- | --- | --- |
| Amino Acids and Derivatives | Thermostable carboxypeptidase | 2 | c(899466..900653) |
|  | Endo-1,4-beta-xylanase | 2 | c(1132682..1134793) |
|  | Adenosylhomocysteine nucleosidase | 2 | 1195076..1195768 |
|  | Uncharacterized HTH-type transcriptional regulator | 2 | 1499991..1500179 |
|  | Transition state regulatory protein AbrB | 2 | 1504211..1504468 |
| Carbohydrates | Glycerol 2-dehydrogenase (NADP(+)) | 2 | 1206521..1207366 |
| Cell Wall and Capsule | N-acetylmuramoyl-L-alanine amidase | 1 | 8329..8475 |
|  | ADP-dependent NAD(P)H-hydrate dehydratase | 2 | 1277054..1277620 |
|  | Probable amino-acid ABC transporter ATP-binding protein | 2 | c(1627055..1627234) |
|  | TPR repeat-containing protein | 2 | 2306203..2307144 |
|  | Probable low-salt glycan biosynthesis flippase Agl15 | 2 | c(2767966..2769438) |
|  | UDP-galactopyranose mutase | 2 | c(2769428..2770540) |
|  | UDP-Glc:alpha-D-GlcNAc-diphosphoundecaprenol beta-1,3-glucosyltransferase WfgD | 2 | c(2775166..2775984) |
|  | Transmembrane protein EpsG | 2 | c(3047193..3048302) |
| DNA Metabolism | UPF0758 protein | 2 | 357974..358423 |
|  | DNA helicase | 2 | 360439..361941 |
|  | Phage-like element PBSX protein XkdC | 2 | 1178027..1178281 |
|  | UPF0758 protein | 2 | 1767581..1767886 |
|  | UPF0758 protein | 2 | c(3475497..3475958) |
| Miscellaneous | Mesentericin-Y105 transport/processing ATP-binding protein MesD | 1 | 2699..4825 |
| Motility and Chemotaxis | Gliomedin | 2 | c(2304840..2305982) |
|  | Collagen alpha-1(IV) chain | 2 | 2431445..2431705 |
|  | Flagellin | 2 | c(2721079..2721885) |
|  | Phosphoribosylformylglycinamidine cyclo-ligase | 3 | c(2663..3184) |
| Nucleosides and Nucleotides | 2',3'-cyclic-nucleotide 2'-phosphodiesterase | 2 | 897814..898494 |
| Phages, Prophages, Transposable elements, Plasmid | Putative DNA recombinase | 2 | c(1495205..1496797) |
|  | Repressor LexA | 2 | c(1499414..1499824) |
| Phosphorus Metabolism | Response regulator ArlR | 2 | 1842152..1842751 |
| Protein Metabolism | Glutamate carboxypeptidase | 2 | 902223..903335 |
|  | Uncharacterized N-acetyltransferase YjaB | 2 | 1193865..1194311 |
|  | Methionine--tRNA ligase | 2 | 1697010..1697213 |
|  | Putative aminopeptidase YhfE | 2 | c(2759040..2760080) |
|  | Tetracycline resistance protein TetP | 2 | c(3486280..3488238) |
| RNA Metabolism | Probable serine/threonine-protein kinase tsuA | 2 | 1159188..1159898 |
| Virulence, Disease and Defense | Mercuric resistance operon regulatory protein | 2 | 1180190..1180984 |
|  | Iron-sulfur flavoprotein | 2 | 1207412..1208047 |
| Not in Subsystem | RTX-III toxin determinant D | 1 | 4815..6326 |
|  | Site-specific DNA-methyltransferase (adenine-specific) | 1 | 16027..16716 |
|  | Probable anaerobic C4-dicarboxylate transporter DcuC | 2 | 900847..902208 |
|  | CAAX prenyl protease | 2 | 1181091..1181915 |
|  | Uncharacterized immunity region protein | 2 | c(1498952..1499401) |
|  | Probable integrase/recombinase YoeC | 2 | 1505742..1506290 |
|  | N-acetylmuramoyl-L-alanine amidase | 2 | 1526757..1527644 |
|  | UPF0702 transmembrane protein YetF | 2 | 1862746..1863054 |
|  | UPF0702 transmembrane protein YetF | 2 | 1863085..1863456 |
|  | dTDP-4-dehydrorhamnose reductase | 2 | c(2772913..2773791) |
|  | Uncharacterized MFS-type transporter YxaM | 2 | c(3488222..3489484) |
|  | Choloylglycine hydrolase | 3 | c(4083..5072) |
